# Sensory neuropathy in amyotrophic lateral sclerosis: a systematic review

**DOI:** 10.1007/s00415-023-11954-1

**Published:** 2023-08-23

**Authors:** Alessandro Bombaci, Antonino Lupica, Federico Emanuele Pozzi, Giulia Remoli, Umberto Manera, Vincenzo Di Stefano

**Affiliations:** 1 “Rita Levi Montalcini” Department of Neuroscience, University of Turin, Turin, Italy; 2 Department of Neuromuscular Diseases, Queen Square Institute of Neurology, UCL, London, WC1N 3BG UK; 3 Department of Biomedicine, Neuroscience and Advanced Diagnostics (BiND), University of Palermo, 90127 Palermo, Italy; 4 Neuroscience, University of Milano-Bicocca, Milan, Italy; 5 Neurology Department, Fondazione IRCCS San Gerardo, Monza, Italy; 6 SC Neurologia 1U, Città della Salute e della Scienza di Torino, Turin, Italy

**Keywords:** Amyotrophic lateral sclerosis, Sensory neuropathy, Systematic review, Neurodegeneration, Non-motor symptoms

## Abstract

Amyotrophic lateral sclerosis (ALS) is a fatal neurodegenerative disease characterized by the degeneration of both upper and lower motoneurons, leading to motor and non-motor symptoms. Recent evidence suggests that ALS is indeed a multisystem disorder, associated with cognitive impairment, dysautonomia, pain and fatigue, excess of secretions, and sensory symptoms. To evaluate whether sensory neuropathy could broaden its spectrum, we systematically reviewed its presence and characteristics in ALS, extracting data on epidemiological, clinical, neurophysiological, neuropathological, and genetic features. Sensory neuropathy can be found in up to 20% of ALS patients, affecting both large and small fibers, although there is a great heterogeneity related to different techniques used for its detection (electromyography vs skin biopsy vs nerve biopsy). Moreover, the association between CIDP-like neuropathy and ALS needs to be better explored, although it could be interpreted as part of the neuroinflammatory process in the latter disease. Sensory neuropathy in ALS may be associated with a spinal onset and might be more frequent in SOD1 patients. Moreover, it seems mutually exclusive with cognitive impairment. No associations with sex and other genetic mutation were observed. All these data in the literature reveal the importance of actively looking for sensory neuropathy in ALS patients, and suggest including sensory neuropathy among ALS non-motor features, as it may explain sensory symptoms frequently reported throughout the course of the disease. Its early identification could help avoid diagnostic delays and improve patients’ treatment and quality of life.

## Introduction

Amyotrophic lateral sclerosis (ALS) is a fatal neurodegenerative disorder characterized by progressive degeneration and loss of function of the motor neurons in the motor cortex (upper motor neuron) and the brainstem and the spinal cord (lower motor neuron) [1]. Approximately 70% of patients have a spinal onset, 30% a bulbar onset, and only a small proportion present with respiratory onset.

Recent studies draw attention to the non-motor symptoms in ALS [2] that significantly reduce the quality of life and can be related to the diffusion of the pathological process to structures such as frontal and temporal cortices, hypothalamus, basal ganglia, and autonomic nervous system. These symptoms include, but are not limited to, cognitive impairment and behavioural alterations (and even full-blown fronto-temporal dementia), pain, fatigue, dysautonomia, sleep problems, sialorrhea, and sensory symptoms [3]. Two recent studies [4, 5] systematically evaluated the non-motor symptoms of ALS, but considered pain and sensory disturbances indistinctly.

Although ALS and sensory neuropathies have been described as separate conditions, they have been considered by some authors as part of the same disorder [6, 7].

The ALS Gold Coast diagnostic criteria [8] require the exclusion of the presence of significant clinical or electrophysiological neuropathy due to other causes [9, 10]; despite that, many authors reported an overlap of both sensory and motor symptoms in ALS patients, usually developed in a time-related sequence. The coexistence of motor and sensory symptoms may delay diagnosis, and the literature on this topic is quite heterogeneous, including clinical, neurophysiological [nerve conduction studies (NCS) from different nerves], and pathological studies (nerves and skin biopsies).

Identifying recurrent neuropathic features in ALS patients could help to improve diagnostic algorithms, identify mixed phenotypes, and ease early diagnosis. Therefore, to clarify and summarize the above-mentioned studies, we conducted a systematic literature review focused on ALS and sensory neuropathy.

## Methods

This systematic review was performed following the principles of the Preferred Reporting Items for Systematic Reviews and Meta-Analyses (PRISMA) 2020 statement [11]*.*

The inclusion criteria were as follows:

- Studies on ALS and any kind of peripheral sensory neuropathy;
- Studies on human subjects, including case reports, case series, cohort studies (prospective or retrospective), cross-sectional and other observational studies;
- Articles written in English

The exclusion criteria were as follows:

- Review articles or meta-analyses, editorials;
- Studies that describe all other forms of motor neuron diseases other than ALS, including the facial onset sensory and motor neuronopathy (FOSMN), already recently described in an extensive and complete review [12].

Review and meta-analysis reference lists were checked to identify additional eligible studies and to elucidate theoretical aspects of the discussion.

We conducted electronic searches for eligible studies within each of the following databases, up to August 14th, 2022: Cochrane Central Register of Controlled Trials (CENTRAL), MEDLINE (accessed via PubMed), Embase, Scopus, and Web of Science. In addition, we checked the reference list of all screened study reports to identify further eligible studies.

Records were identified in each database with the following search strings on MEDLINE: *“Amyotrophic Lateral Sclerosis”[Mesh]) AND (“Polyneuropathies”[Mesh] OR “Peripheral Nervous System Diseases”[Mesh]*, and with the following string on the other databases: *“Amyotrophic Lateral Sclerosis” AND “peripheral neuropathy”.*

No additional filters were used to maximize sensitivity.

Citations identified from the literature searches were imported to Mendeley and duplicates were automatically removed by the software. Then, all reviewers independently screened the records’ titles and abstracts. In case of disagreements about eligibility, a consensus was reached through discussion. The full texts of all potentially eligible studies, reviews, and meta-analyses were retrieved, and their reference lists were checked. All reviewers performed data extraction from eligible studies. We extracted data about authors, year, country, study design, number of participants, gender, age (or mean age), duration of follow-up, diagnostic criteria for ALS and neuropathy, type of neuropathy, autoptic results, genetics, symptoms and type of onset, neurophysiological exams, other exams, dysautonomia, comorbidities, therapy, and limitations.

Since the identified studies were highly heterogeneous, no meta-analysis could be performed, which could limit the strength of the conclusions reported in our paper. In the results section we will provide an overview of the case reports and observational studies, together with a quantitative synthesis. Weighted means are reported in the observational studies section for age, age at onset, and time to evaluation. Absolute percentages are reported for categorical variables. Comparisons between groups have been performed with t-tests for continuous variables. Case reports will be analyzed separately since they provided types of information that did not overlap with observational studies. Then, in the discussion, we will provide an interpretation of the clinical features, neurophysiological characteristics, neuropathological findings, and genetics of ALS with sensory neuropathy.

## Results

**Fig. 1:**
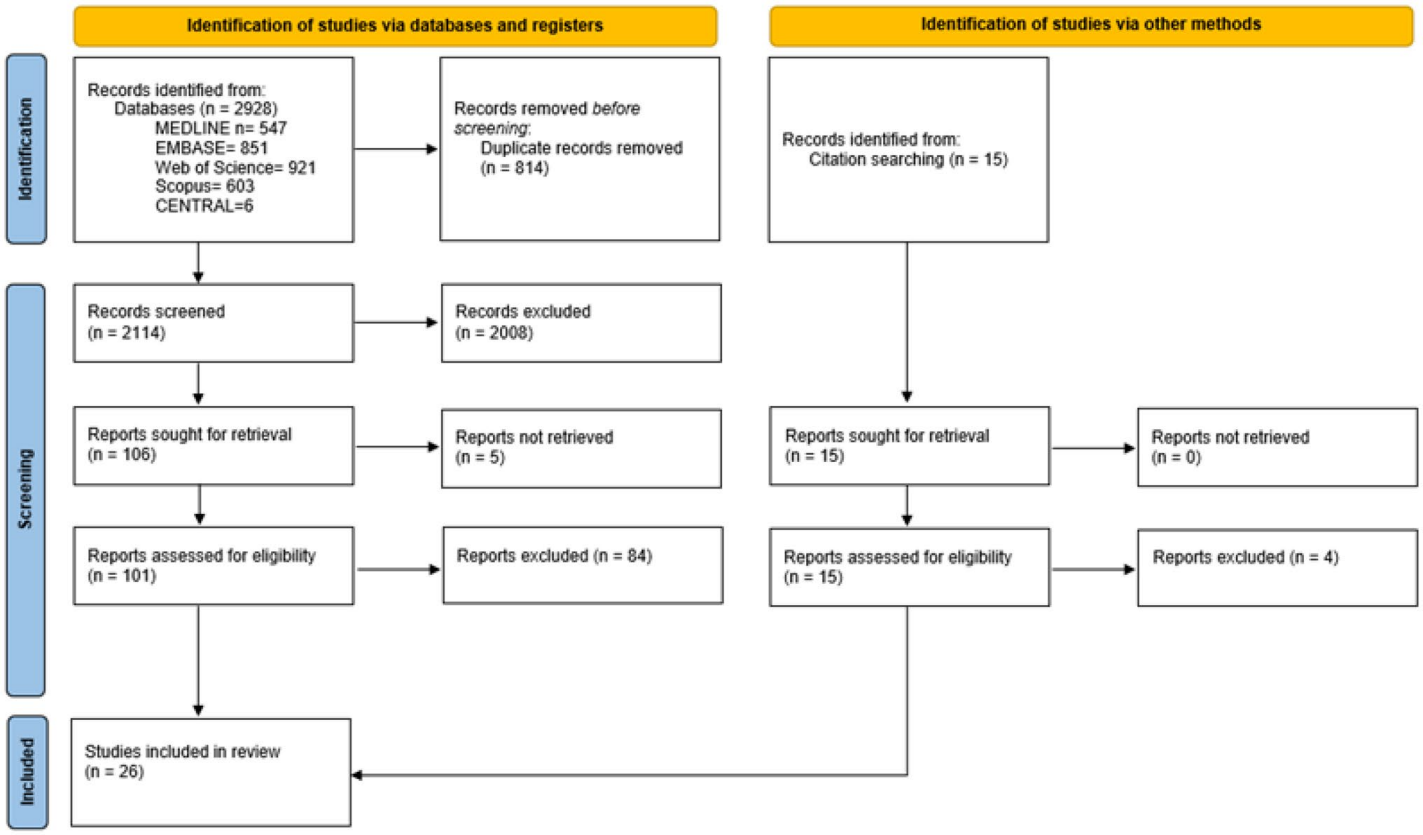
PRISMA flowchart of the included studies

We identified 2928 records through the initial search. After duplicate removal, 2114 titles were screened, and 2008 were excluded. Of the remaining 106 reports, 5 could not be retrieved due to the unavailability of the full text. 101 abstracts were retrieved and assessed for eligibility. Of these, 84 were excluded because either they did not provide enough data to evaluate the risk of bias, did not meet the other inclusion criteria, or met one of the exclusion criteria. We added other 11 reports checking the reference lists of the retrieved studies. Finally, 28 studies were included in this review (the flow diagram is shown in Fig. 1).

### Case reports and case series

A total of 16 case reports were included, encompassing 29 patients. Of these, 21 had ALS and sensory neuropathy, 3 had ALS and hereditary neuropathy with liability to pressure palsies (HNPP, with an additional case described in an observational study), 3 had juvenile ALS with neuropathy (one of these cases was described as an abstract at a congress, and another case described in an observational study), one had ALS and Charcot-Marie-Tooth 2A (CMT2A), and one had ALS and complex painful regional syndrome type I (CPRS, without signs of nerve damage). The patients included in the case reports are summarized in Table 1. Except for a case described by Nishijima, who had diabetes and developed a chronic inflammatory demyelinating polyneuropathy (CIDP)-like demyelinating neuropathy [13], no other patient had other known causes of sensory neuropathy (such as diabetes, vitamin B12 deficiency, history of alcohol abuse, amyloidosis, cancer, or chemotherapy).

**Table 1:** Case reports

						ALS				Neuropathy					
References	Sex	Age	Nation	Last FU	FU	Onset	Genetics	Type	TAD	Genetics	Criteria	Type	ACD	Therapy	Effectiveness
Akaishi et al. [14]	F	37	Japan	Dead	11.9	SN	NP	Spinal	3	PMP22-	EFNS/PNS	Demyelinating	NR	IVIG, oral steroids	±
Nishiyama et al. [26]	F	51	Japan	Alive	14	SN	SOD1 L8V	Spinal	4	Negative	EMG	Axonal	NR	None	NA
Wakabayashi et al. [16]	M	57	Japan	Dead	4.3	SN	NP	Spinal	2	NP	NR	Demyelinating and axonal	Yes	Steroids, PLEX	–
Echaniz-Laguna et al. [15]	M	44	France	Dead	1.6	SN	SOD1-	Spinal	1	NP	AAN	Demyelinating	Yes	IVIG, steroids	NR
Echaniz-Laguna et al. [15]	M	63	France	Dead	1	SN	SOD1-	Spinal	0.5	NP	AAN	Demyelinating	Yes	IVIG	NR
Echaniz-Laguna et al. [15]	M	52	France	Dead	3.1	SN	NP	Spinal	1	NP	AAN	Demyelinating	Yes	IVIG, steroids, and cyclophosphamide	–
Nishijima et al. [13]	M	45	Japan	Alive	4	SN	NP	Spinal	1	NP	EFNS/PNS	Demyelinating	NR	IVIG, steroids, PLEX	–
Nishijima et al. [13]	M	68	Japan	Dead	1.2	SN and ALS	NP	Spinal	0	NP	EFNS/PNS	Demyelinating	Yes	IVIG, steroids, PLEX	±
Rajabally et al. [18]	F	65	UK	Alive	2.8	SN	NP	Spinal	1	NP	EFNS/PNS and AAN	Axonal (pure motor)	NR	IVIG	±
Rajabally et al. [18]	M	65	UK	Alive	2.5	SN	NP	Spinal	1.5	NP	EFNS/PNS and AAN	Axonal	NR	IVIG	–
Rajabally et al. [18]	F	57	UK	Alive	6.5	SN	NP	Spinal	3.5	NP	Nicholas	Axonal	NR	IVIG	–
Rajabally et al. [18]	F	68	UK	Dead	2.6	SN	NP	Flail arm	2	NP	EFNS/PNS and AAN	Axonal and demyelinating	Yes	Steroids, IVIG, PLEX	± (only IVIG)
Rezania et al. [32]	M	69	USA	Alive	4.9	SN and ALS	SOD1 (A89V)	Spinal	1	NP	EMG	Axonal	NR	NR	NR
Sawa et al. [27]	M	66	Japan	Alive	5	SN	NP	Spinal	NR	NP	EFNS/PNS	Demyelinating	Yes	IVIG, steroids	–
Sawa et al. [27]	F	53	Japan	Alive	4	ALS	NP	Spinal	NR	NP	EFNS/PNS	Demyelinating	Yes	IVIG	±
Sawa et al. [27]	M	50	Japan	Alive	4.6	ALS	NP	Spinal	NR	PMP22-	EFNS/PNS	Demyelinating	Yes	IVIG	–
Isaacs et al. [17]	M	63	UK	Dead	2	ALS	NP	Spinal	NR	NP	EMG	Demyelinating and axonal	Yes	Steroids	–
Isaacs et al. [17]	M	65	UK	Dead	4.6	SN	NP	Spinal	4	NP	EMG	Axonal	No	None	NA
Isaacs et al. [17]	M	49	UK	Alive	10	ALS	NP	Spinal	NR	NP	EMG	Axonal	Yes	None	NA
Isaacs et al. [17]	M	58	UK	Alive	4.5	SN	NP	Flail arm	0	NP	EMG	Axonal	Yes	Steroids	–
Isaacs et al. [17]	M	64	UK	Dead	2.5	SN and ALS	NP	Spinal	NR	NP	NR	Axonal	Yes	Chlorambucil	–
Canali et al. [35]	F	32	Italy	Alive	2	SN	Negative	Spinal	1	PMP22 +	EMG	Demyelinating	Yes	None	NA
O’Sullivan et al. [36]	F	51	Irel&	Alive	0.8	SN and ALS	NP	Spinal	0.75	PNP22 +	EMG	Demyelinating and axonal	Yes	None	NA
Bhatt et al. [34]	M	56	USA	Alive	0.3	SN	Negative	Spinal	0	PMP22 +	NR	Demyelinating	Yes	NR	NR
Marchesi et al. [31]	F	62	Italy	Alive	NR	SN	Negative	Spinal	2	MFN2 +	EMG, Genetics	Axonal	Yes	NR	NR
Ricciardi et al. [44]	M	64	Italy	Alive	0.8	ALS	NP	Spinal	0	NP	IASP 2010	NP	NR	Deflazacort and neridronate	+
Saiga et al. [28]	M	35	Japan	Alive	NR	SN	SETX (c.6406C > T)	Spinal	NR	NP	Clinical	Demyelinating	NR	IVIG, steroids	±
Johnson et al. [25]	F	11	USA	Alive	1	ALS	SPTLC1 (p.Ser331Tyr)	Spinal	7	NP	EMG	Axonal	NR	NR	NR
Tateishi et al. [37]	M	37	Japan	Alive	NR	SN	SETX (R2136C)	Spinal	NR	NP	EMG	Demyelinating	NR	Steroids	±

*AAN* American Academy of Neurology, *ACD* albuminocytological dissociation, *ALS* amyotrophic lateral sclerosis, *EFNS/PNS* European Federation of Neurological Societies/Peripheral Nerve Society, *FU* follow-up (in years), *IASP* International Association for the Study of Pain, *IVIG* intravenous immunoglobulins, *MFN2* mitofusin 2, *NA* not applicable, *NP* not performed, *NR* not reported, *PLEX* plasma exchange, *PMP22* peripheral myelin protein 22, *SETX* senataxin, *SN* sensory neuropathy, *SOD1* super-oxide dismutase 1, *SPTLC1* serine palmitoyltransferase long chain base subunit 1, *TAD* time from presentation with ALS-compatible symptoms to ALS diagnosis (years)

Among the 21 cases with ALS and sensory neuropathy, 9 cases have been described in the UK, 8 in Japan, 3 in France, and one in the US. All subjects had spinal onset ALS, with 2 patients showing the flail-arm pattern; there were no bulbar onset cases. 4 cases had an autoptic ALS confirmation, with degeneration of anterior horn neurons with Bunina bodies and TDP43-immunoreactive cytoplasmic inclusions [13–16]. ALS was clinically diagnosed in all cases based on El Escorial 1994 or 2000 criteria, except in two cases, where the diagnosis was made through autopsy [14, 16].

Either nerve biopsy or autopsy confirmed the neuropathy in most cases (14). Still, there was significant heterogeneity in diagnostic criteria, with 13 cases diagnosed based on EFNS/PNS, AAN, or Nicholas 2002 criteria for CIDP. In the remaining cases, diagnostic criteria were either electromyographical or neuropathological evidence of neuropathy or not stated.

The mean age at either ALS or neuropathy onset was 57.6 ± 9.2 years, not significantly differing between sporadic and presumably familial cases. The mean disease duration of the sporadic cases was 53.9 ± 32.9 months, ranging from 24 to more than 142 months, and probably slightly higher than the mean duration of ALS (around 24–48 months) [1], although only 3 patients significantly exceeded 5 years of ALS duration[14, 17, 18]. After excluding these exceptionally slow progressors, the mean duration of disease was 41.3 ± 13.9 months (range 24–59), overlapping with the expected ALS lifespan.

None of the between-groups differences was significant (see Table 2), but there was a tendency towards shorter times to death in familial vs sporadic ALS cases (*p* = 0.05). However, when all cases (dead or alive) were considered, no significant difference in disease duration between the two groups emerged.

**Table 2:** Analysis of case reports

Variable	All cases	Groups (*n* of patients)	Group 1	Group 2	*p*
Age at onset	57.6 ± 9.15	FALS (4) vs SALS (17)	56.7 ± 11.3	57.7 ± 8.9	0.43
Follow-up (months)	56 ± 40.7	Dead (9) vs Alive (12)	41.2 ± 39.9	67.1 ± 39.2	0.08
Follow-up (months)	56 ± 40.7	FALS (4) vs SALS (17)	64.6 ± 71.6	53.9 ± 33.0	0.39
Follow-up (months)	56 ± 40.7	SALS (17) vs non-slow SALS (14)	53.9 ± 33.0	41.3 ± 13.9	0.08
Follow-up (months)—dead only	41.2 ± 39.9	FALS (2) vs SALS (7)	16.5 ± 4.9	48.3 ± 43.1	0.05
Follow-up (months)—alive only	67.1 ± 39.2	FALS (2) vs SALS (10)	113 ± 77.8	57.9 ± 25.5	0.25
Time to diagnosis (years)	1.7 ± 1.3	FALS (4) vs SALS (11)	1.6 ± 1.6	1.7 ± 1.3	0.46
Time to diagnosis (years)	1.7 ± 1.3	NP first (13) vs ALS/NP + ALS first (2)	1.9 ± 1.3	0.5 ± 0.7	0.07

*FALS* familial ALS, *SALS* sporadic ALS

**Fig. 2:**
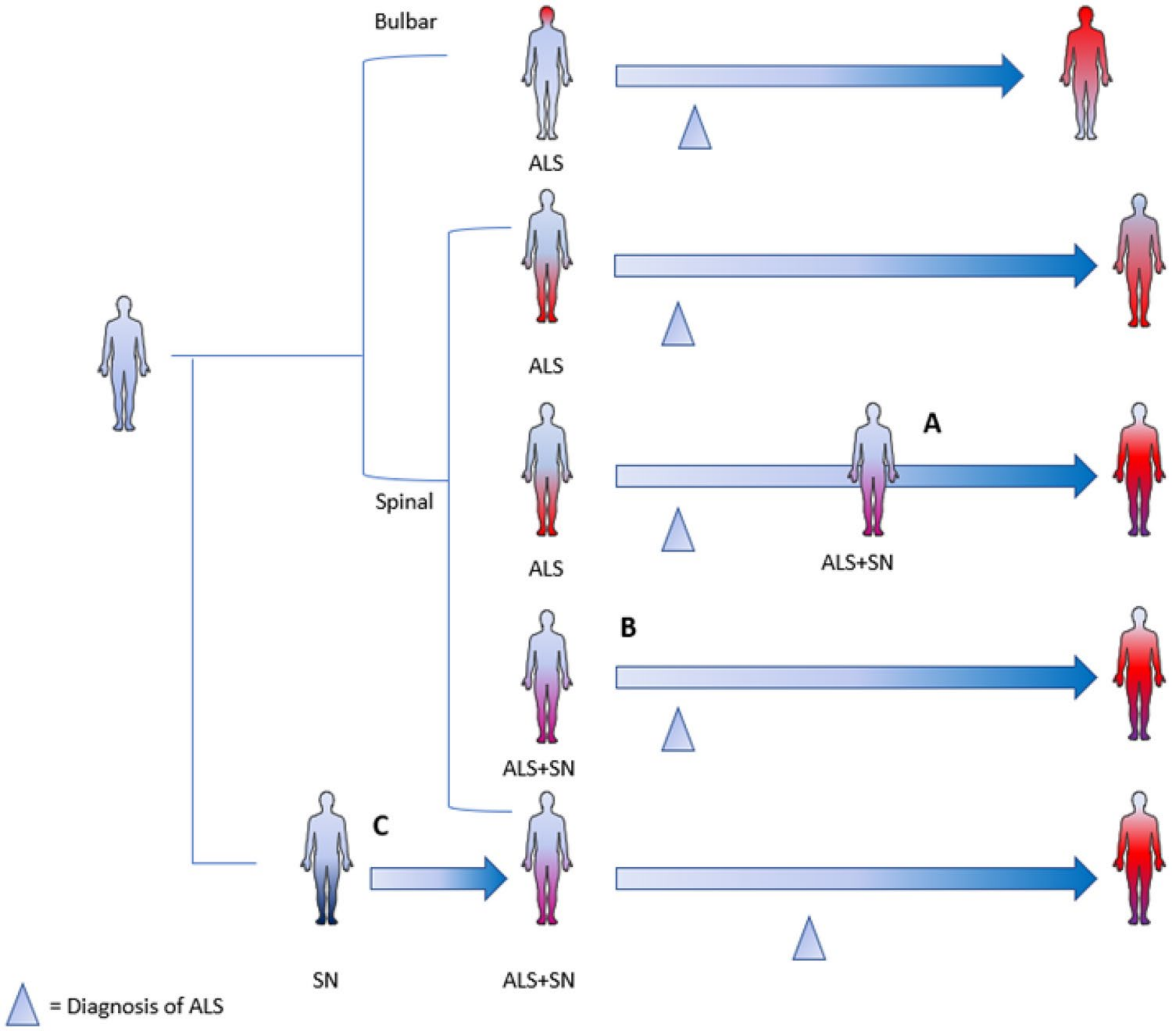
Timeline of ALS and sensory neuropathy. In this figure we can observe the temporal correlation between the onset of ALS and of sensory neuropathy: in spinal onset ALS patients neuropathy could appear before (**C**), after (**A**) o together (**B**) with ALS symptoms. If sensory neuropathy precedes ALS there is usually a delay in ALS diagnosis. Sensory neuropathy does not seem to be associated with bulbar onset of ALS. SN Sensory neuropathy

ALS diagnostic delay was 20.4 ± 16.1 months (range 0–48), not differing between sporadic and presumably familial cases. This is slightly higher than the typical time to diagnosis (usually estimated in 10–16 months [19]). The diagnostic delay might have been driven by the cases with neuropathy preceding ALS (22.6 ± 16.1 months when neuropathy preceded ALS vs 6 ± 8 months when ALS and neuropathy onsets were coincidental, *p* = 0.07). This probably reflects the diagnostic challenges associated with pre-existing neuropathy. A graphical summary of these findings is presented in Fig. 2.

Around 63% of patients were male (15/24), consistent with the fact that ALS is 20% more common in men [20].

### Observational studies

We included a total of 12 observational studies (summarized in Table 3). The study by Bradley focused only on pathological data and will be treated separately [21].

**Table 3:** Observational studies

References	Type	ALS pts	Sex (F, %)	Age (mean)	Nation	Spinal ALS	SN	SN criteria	SN type	AO	TE	SS	CI
Weis et al. [29]	Cross-sectional	28	8 (28)	58	Germany	23	22	Skin biopsy	Small fiber	54.9	2.8	7	NR
Truini et al. [40]	Cross-sectional	24	8 (33)	65.2	Italy	13	11	Skin biopsy	Small fiber	NR	2.2	6	Excluded
Mondelli et al. [30]	Cross-sectional	64	38 (59)	58.8	Italy	52	14–32	EMG	Axonal	NR	1.6	0	NR
De Carvalho et al. [39]	Longitudinal cohort study	339	148 (44)	NR	Portugal	243	29	EMG	Axonal	61.5	1.75	0	5
Gregory et al. [6]	Longitudinal cohort study	12	4 (21)	57	UK	12	0	NR	Axonal	58.1	0.9	2	NR
Pugdahl et al. [9]	Retrospective cohort study	88	40 (45)	63.6	Europe	61	12	Consensus diagnosis (Clinical + EMG)	Axonal and/or demyelinating	NR	0.8	0	NR
Hammad et al. [7]	Retrospective cohort study	103	39 (38)	59	USA	103	20 (sural biopsy) or 28 (EMG)	EMG or sural biopsy	Axonal (CV not performed)	NR		33	7 (ALS-FTD)
Luigetti et al. [38]	Retrospective cohort study	17	1 (6)	61.1	Italy	16	12	Sural biopsy	Axonal; one case axonal and demyelinating (with inflammatory infiltrate), one case HNPP	NR	2.7	5	NR
Ahlskong et al. [22]	Cross-sectional	7 (ALS, PDC or PDC + ALS)	NR	NR	Guam	NR	4	EMG or clinical	Axonal	NR	NR	NR	3 (PDC)
Devigili et al. [23]	Retrospective study	18	7 (38)	58.6	Germany	NR	11	Sural biopsy	Vasculitis-like infiltrates	NR	NR	NR	NR

*ALS* amyotrophic lateral sclerosis, *AO* Age at onset (mean), *CI* patients with cognitive impairment, *CV* conduction velocity studies, *F* females, *FTD* fronto-temporal dementia, *NR* not reported, *PDC* Parkinson-dementia complex, *SN* sensory neuropathy, *SS* patients with sensory signs or symptoms, *TE* Time to evaluation (in years, mean)

One of these studies evaluated sensory neuropathy in Chamorro patients with either ALS or Parkinson-dementia and ALS complex (PDC-ALS), a still obscure tauopathy found among a particular ethnic group on the Pacific island of Guam [22]. Four out of 7 patients also showed an (axonal) neuropathy, defined by clinical (2) or neurophysiological means (2), with a distal–proximal severity gradient. One of these patients had PDC without ALS, suggesting that the neuropathy may be yet another primary feature of this exceptionally broad neurodegenerative disease, which may exhibit variably combined phenotypes. However, 3 patients with neuropathy also had diabetes, which may limit the validity of these conclusions [22].

The remaining papers, all from first-world countries, included a total of 728 patients, with 310 females (43%). The mean age was 60.9 years, with a mean age at onset of 61.4 years. This is explained by the fact that only four papers including a total of 467 patients reported age at onset; of these, three also reported mean age at the time of evaluation. In these three papers (including 379 patients), the mean age at evaluation was 61.1, while the mean age at onset was 60.9. Summary statistics for these studies can be found in Table 4; the study by Devigili selected the ALS sample according to criteria that were different from the remaining papers and was not included in the numerical analysis [23].

**Table 4:** Observational studies summary statistics

SN definition	ALS pts	Females (%)	Age (mean)	Age at onset	Spinal onset (%)	SN (%)	SS (%)	TE (years)
All	675	286 (42)	60.9	61.4	523 (77)	128 (19)	53 (8)	1.6
EMG/clinical	623	270 (43)	61.0	62.1	487 (78)	95 (15)	40 (6)	1.5
Skin biopsy	52	16 (31)	61.3	54.9	36 (69)	33 (63)	13 (25)	2.8
Sural biopsy	39	10 (26)	57.7	NR	38 (97)	32 (82)	12–17 (31–44)	2.4
All biopsies	91	26 (29)	59.8	54.9	74 (81)	65 (71)	25–30 (27–33)	2.6

*ALS* amyotrophic lateral sclerosis, *NR* not reported, *TE* Time to evaluation (in years, mean), *SN* sensory neuropathy, *SS* patients with sensory signs or symptoms

It was not possible to determine whether ALS came before neuropathy or vice versa, either because this information was not reported, or because exclusion criteria eliminated cases in which neuropathy predated ALS onset, resulting in a loss of cases of ALS with superimposed sensory neuropathies.

554 patients had a spinal onset of ALS (76%), although two studies excluded patients with bulbar onset [6, 7].

Age was confirmed as a small risk factor for neuropathy in ALS in two large EMG studies, as each additional year was found to increase the risk of polyneuropathy by 1.044 [9, 24]. Conversely, the female gender decreased the risk of polyneuropathy by 0.644, while respiratory onset increased the risk of polyneuropathy by 5.4 [24].

### Clinical features of patients with ALS and sensory neuropathy

Among the 29 cases described in case reports, 20 patients (69%) reported sensory signs or symptoms. Looking at the 728 ALS patients reported in the 10 observational studies included in this review 142 subjects (19.5%) were affected by neuropathies and 55 (7.6%) had sensory clinical signs or symptoms. However, at least three different ways of assessing neuropathy have been proposed, namely with a skin biopsy (for small fiber neuropathy), sural biopsy, and EMG. These different definitions resulted in different prevalences of neuropathy, 63.4%, 82.0%, and 16.3% respectively. Sensory symptoms or signs were most prevalent in the sural biopsy group, ranging between 30.7% and 43.5%, and in the skin biopsy group (25%) compared to the EMG group (6.4%).

Signs and symptoms usually reported are tingling and deep and superficial hypoesthesia. In 2 cases patients referred pain and allodynia in the lower limbs.

Sensory signs and/or symptoms seem to have a distal–proximal gradient and are more frequently reported in the lower limbs. In 31 patients sensory symptoms were also distributed in the upper limbs [7, 13, 14, 16, 18, 25–28]. Only one patient described in an observational study [29] reported facial symptoms and one patient in another observational study [29] reported symptoms at the level of the trunk.

In the case reports and the case series, we were able to look at the correlation between the onset of sensory clinical signs and/or symptoms and the onset of motor signs and/or symptoms. We highlighted 3 different scenarios: the first in which the motor impairment precedes the sensory one (4 out of 20 ALS patients), the second in which the sensory impairment precedes the motor one (4 out of 20 ALS patients), and the third and most frequent, in which there is a concomitant onset (12 out of 20 ALS patients).

In the first and in the second scenarios the reported delay between sensory and motor signs and/or symptoms was between 6 and 48 months.

For patients included in observational studies, this type of analysis was not possible because it was not available a detailed report of the timing of the onset of sensory and motor signs and/or symptoms for each patient. Nevertheless, it was evident that sensory clinical alterations could begin before, after, or together with motor ones.

Focusing on the ALS onset, among the 728 ALS patients included in this review no ALS patient with a bulbar onset presented sensory clinical signs and/or symptoms.

Moreover, sensory signs and/or symptoms described in ALS patients with known genetic mutation did not differ from the ones described by all the other sporadic ALS patients.

Focusing on ALS patients with cognitive impairment, none of them had associated sensory signs and/or symptoms. However, only a reduced number of ALS patients underwent neuropsychological tests.

### Neurophysiological findings in ALS

Even though NCS has not been performed in all included studies, most studies reported sensory-motor axonal neuropathy (Table 5). Reduced or absent responses from motor conduction studies of distal muscles are a mainstay in ALS because of degeneration of the anterior horn cells [6, 17, 18, 22, 24–26, 30–32]. Most studies indicate little or no change in motor conduction velocity due to the preservation of the fastest fibers for a long time [33]. However, although a slight slowing of motor conduction is expected in ALS, reflecting a minimal loss in fast fibers, demyelinating motor neuropathy features with prolonged distal motor latency, slowing of conduction velocities, conduction blocks, and prolonged F-waves latencies have also been described isolated [13–15, 27, 34, 35] or combined with axonal damage [9, 16–18, 28, 36–38].

**Table 5:** Neurophysiological features of neuropathy detected on nerve conduction studies and electromyography in ALS patients

	Neurophysiological features	References
Sensory conduction studies of the upper limbs (median, ulnar, radial nerve)	Reduced or absent SAP with reduction of conduction velocity	Gregory et al. [6], Mondelli et al. [30], Wakabayashi et al. [16], O’Sullivan et al. [36], Isaacs et al. [17], Bhatt et al. [34], Canali et al. [35], Tateishi et al. [37]
Sensory conduction studies of the lower limbs (sural nerve)	Reduced or absent SAP with reduction of conduction velocity	Gregory et al. [6], Wakabayashi et al. [16], Rezania et al. [32], Echaniz-Laguna et al. [15], Hammad et al. [7], Isaacs et al. [17], Pugdahl et al. [9], Rajabally et al. [18], Weis et al. [29], Luigetti et al. [38], Truini et al. [40], Nishiyama et al. [26], De Carvalho et al. [39], Johnson et al. [25]
Motor conduction studies of the upper limbs (median, ulnar, radial nerve)	Reduced or absent CMAPs amplitudes	Mondelli et al. [30], Gregory et al. [6], Ahlskong et al. [22], Rezania et al. [32], Isaacs et al. [17], Rajabally et al. [18], Marchesi et al. [31], Nishiyama et al. [26], De Carvalho et al. [39], Johnson et al. [25]
	Prolonged DML, slowing of motor conduction velocities, conduction blocks, and prolonged F-waves latencies	Echaniz-Laguna et al. [15], Bhatt et al. [34], Canali et al. [35], Nishijima et al. [13], Sawa et al. [27], Akaishi et al. [14]
Motor conduction studies of the lower limbs (peroneal, tibial nerve)	Reduced or absent CMAPs amplitudes	Mondelli et al. [30], Gregory et al. [6], Ahlskong et al. [22], Rezania et al. [32], Isaacs et al. [17], Rajabally et al. [18], Marchesi et al. [31], Nishiyama et al. [26], De Carvalho et al. [39], Johnson et al. [25]
	Prolonged DML, slowing of motor conduction velocities, conduction blocks and prolonged F-waves latencies	Echaniz-Laguna et al. [15], Bhatt et al. [34], Canali et al. [35], Nishijima et al. [13], Sawa et al. [27], Akaishi et al. [14]
Electromyography of the upper and lower limbs	Fasciculation potentials, positive sharp waves, enlarged and polyphasic MUPs with increased duration	Echaniz-Laguna et al. [15], O’Sullivan et al. [36], Bhatt et al. [34], Marchesi et al. [31], Nishijima et al. [13], Sawa et al. [27], Isaacs et al. [17], Ricciardi 2020, Johnson et al. [25]

*SAP* Sensory action potentials, *CMAP* compound motor action potential, *DML* distal motor latency, *MUP* motor unit potential

It is worth noting that subtle abnormality in sensory action potential (SAPs) may be detected in some ALS patients. In particular, a reduced or absent SAP with a reduction of conduction velocity in the sural nerve [6, 7, 9, 15–18, 25, 26, 29, 32, 38–40] or the upper limbs was observed in ALS patients [6, 16, 17, 30, 34–37].

Among the 24 ALS patients with associated sensory neuropathies described in the cases reports, excluding the 3 ALS with associated HNPP, CMT2A, and CPRS, in 11 cases the neuropathy was predominantly demyelinating, in 10 cases predominantly axonal, and mixed demyelinating and axonal in the remaining 3 cases. In most of the demyelinating and mixed neuropathies, the criteria for CIDP were met [13–15, 18, 27]. Interestingly, in most cases of ALS associated with CIDP-like neuropathies no autoimmune comorbidity was reported, except for a case of MGUS [17].

Looking at observational studies in which electromyography was performed, sensory neuropathy was almost always axonal and mild and worsening over time, supporting the notion of a parallel neurodegenerative axonopathy as a part of ALS pathophysiological spectrum [6, 30]. Axonal sensory neuropathy was described in 16.3% of ALS patients investigated, although it is not entirely associated with clinical correlates. Indeed, such a neuropathy could be asymptomatic, and, sometimes, sensory complaints may not necessarily be the result of underlying nerve damage. For instance, in the series described by Hammad, among the 28 patients with EMG evidence of neuropathy, only 10 were symptomatic, while the remaining 23 symptomatic patients did not show EMG evidence of neuropathy [7].

Somatosensory evoked potentials (SSEPs) in ALS can display delayed peripheral sensory conduction or absent responses from stimulation of the tibial nerve at the lumbosacral and primary sensory cortex [26, 28]; also, SSEPs latencies from stimulation of the median nerve might be increased [6]. Motor-evoked potentials (MEPs) from both upper and lower limbs might display initially an asymmetric prolongation of the central and peripheral conduction time and a high threshold for cortical stimulation, suggesting cortical hypoexcitability at rest [28, 35]. In advanced stages, a significant reduction or absence of responses seems to be the rule [41].

### Neuropathological findings

Among the articles reviewed, histopathological data focused on the research of neuropathic sensory involvement are reported in 16 case reports and in 6 observational studies. In other cases, described histopathological findings are used only to confirm the diagnosis of ALS.

The presence of a neuropathic sensory involvement in ALS mainly derives from skin and/or nerve biopsies [7, 23, 29, 38, 40], although there is also a post-mortem study [21].

Both large and small sensory fibers seem to be involved. Weiss [29] and Truini [40] reported a distal sensory small fiber degeneration, resulting in a reduction of intraepidermal nerve fiber density (IENFD) in skin biopsies of ALS patients, compared to healthy controls. Weiss also reported a reduction of sweat gland innervation and subepidermal nerve plexus density in the distal calf in ALS compared to controls [29]. The involvement of intraepidermal sensory fibers is supported by the alteration of Quantitative Sensory Testing (QST): 11 out of 13 ALS patients described by Truini showed increased warm detection thresholds and reduced cold detection thresholds when compared with the reference ranges [40]. Looking at the site of disease onset, Truini highlighted that ﻿IENFD appeared spared in all 11 bulbar onset ALS, in contrast with the 13 ALS with spinal-onset, among which 11 cases showed a significantly reduced IENFD [40]. The authors reported that the most likely explanation for this could be that sensory system damage follows motor involvement. Therefore, they highlighted that an involvement of sensory fibers of the cranial nerves in patients with bulbar-onset ALS could not be excluded, given that a biopsy in such regions was not performed. Anecdotally, Wakabayashi described a case report of an ALS patient with moderate neuronal loss in the facial and hypoglossal nuclei, and loss of myelinated fibers in the trigeminal, facial, and glossopharyngeal nerves [16].

Large fiber involvement has been reported in studies on sural nerves biopsy [7, 21, 23, 38]. Bradley et al. reported that the sural nerves of 21 patients with ALS showed a significantly higher percentage of fibers undergoing acute axonal degeneration and 30% fewer myelinated fibers than controls [21]. In another study, a sural nerve biopsy revealed the involvement of sensory fibers in 70–91% of ALS patients [7, 38]. Demyelination was observed in a significant portion of subjects on sural biopsies, with large-caliber myelinatedfibers being more affected than small-caliber myelinated fibers [7, 38].

In some cases, inflammatory features appeared in ALS sural samples [23, 38]. In particular, Devigili et al. reported in 11/18 ALS patients with sensory symptoms the presence of dense epineural perivascular T cell infiltrates indistinguishable from vasculitis nerve exchange. Similar observations are available from a case reported by Luigetti in which neurophysiological abnormalities similar to that typical of inflammatory neuropathies were observed [38].

Histopathological data of sensory neuropathy in ALS are also described in 16 case reports [13, 14, 16–18, 26–28, 32, 34, 37], in which a sural nerve biopsy was performed. In these cases, both a reduction of the number of fibers and damage to myelinated fibers are observed.

4 patients reported in case reports underwent autopsies [13–16], which show a clear loss of nerve fibres in the dorsal nerve roots associated with the presence of thinly myelinated fibres.

### Genetics

Approximately 15% of ALS patients report a positive family history, typically with dominant inheritance [42]. C9Orf72 expansion is the most common form of hereditary ALS in Europe, North America, and Australia, while SOD1 mutation is the second in Europe and the first in Asia [42]. Among studies included in this review, genetic testing was performed only in a minority of cases.

In case reports genetics of ALS was performed in only four patients [15, 26, 32]; in two cases, a SOD1 mutation was found, while in other two cases, SOD1 was negative (but the patients had a brother and a son respectively with typical ALS, raising the suspicion of familial ALS [15]). Another case reported by Weiss in an observational study carried a SOD1 mutation (H48R) and reported sensory disturbances [29], while other three patients with SOD1 mutations reported by Luigetti and De Carvalho did not [38, 39]. In the case described by Nyshiyama (patient 2 [26]), a SOD1 L8V mutation was found, with an exceptionally long disease duration, the patient being alive at least 10 years after probable ALS onset and 14 years after possible axonal neuropathy onset. The case described by Rezania carried a SOD1 A89V mutation, and was alive at 5 years of follow-up, although wheelchair-bound. He had coexisting spinal onset ALS and axonal neuropathy. The same mutation produced heterogeneous disease paths in his family, with a first-degree cousin without neuropathy, but with sensory signs (tingling at the toes), an asymptomatic son at 44, and a grandson with juvenile ALS onset (at 15 years) and no sensory signs or neuropathy [32]. The other two patients with possible SOD1-negative familial ALS had a rather aggressive disease course, with coexisting CIDP-like neuropathy beginning before ALS, no response to IVIG therapy, and death at 13 and 20 months from onset respectively [15].

The association between motor and sensory symptoms has been also investigated in C9Orf72 ALS patients [24, 43]. Although a limited number of patients have been included in these analyses, no differences have been observed compared to sporadic ALS patients.

Three cases of juvenile ALS with sensory neuropathy have been included, all with spinal onset ALS and dysautonomia (neurogenic bladder in the first two cases [28, 37], postural tachycardia and hyperhidrosis in the other [25]). The first patient was a 35 years-old Japanese male, who carried a heterozygous c.6406C > T SETX mutation. In this case a motor demyelinating neuropathy with adjunctive sensory features (sensory disturbances, delayed sensory conduction on SSEP, and mild sural demyelination on biopsy) predated ALS of 6 years, and was partially responsive to IVIG cycles [28]. Indeed, a similar case had been already described by Tateishi, with a distinct mutation in SEXT (R2136C) [37]. Finally, Johnson reported the case of an 11 years old female with ALS carrying SPTLC1 p.Ser331Tyr mutation, who later developed an axonal sensory neuropathy. Interestingly, other reported patients with different SPTLC1 mutations linked to juvenile ALS did not show not any sensory involvement [25]. Curiously, an additional case carrying a TDP43 p.A382T mutation described by Luigetti in an observational study presented a mild axonal loss on sural nerve biopsy, but normal ENG parameters; the association of this TDP43 mutation with peripheral neuropathy has been previously reported in another patient without ALS [38].

Three cases with coincidental ALS and genetically-confirmed HNPP were reported, all with spinal onset and demyelinating neuropathy [34–36]. In two cases the onset of the neuropathy predated the onset of ALS, while in one case they were presumably simultaneously. Age at presentation for ALS ranged from 32 to 56 years. An additional case was reported by Luigetti in an observational study, with not enough information to compare it to the others listed here [38].

Only one case reported an association between ALS, with onset at 62 years, and a long-standing neuropathy due to genetically-confirmed CMT2A.

Ricciardi described a case of a 64 years-old male from Italy with spinal onset ALS developing CPRS-I, which was significantly improved by the administration of deflazacort and neridronate [44].

### Treatment

No observational study or randomized controlled trial on therapies was available, and data derive only from case reports and case series. Most ALS patients affected by sensory neuropathies were treated with a course of IVIG, with only partial and transient responses in a few patients [14, 18, 27]; on the other hand steroids, plasma exchange, and chlorambucil were all ineffective [13, 15–18, 27, 37]. Patients who responded transiently to IVIG mainly presented CIDP-like features.

## Discussion

This systematic review provided an up-to-date qualitative and quantitative analysis of the prevalence of sensory system impairment in ALS patients and its features.

By definition, neuroscientists consider that sensory fibers are not damaged in ALS and the presence of sensory symptoms always needs to be better defined and studied since it could be explained by other causes [20]. Nevertheless, many papers on ALS included in our review report a coexistence of both sensory and motor symptoms, timely related to ALS, and apparently not due to other causes, and the presence of sensory neuropathy in a significant proportion of ALS patients. Therefore, sensory neuropathy should be included in the list of non-motor symptoms of ALS.

From a clinical point of view, it is fundamental to be aware of the possible involvement of sensory systems in ALS in order not to delay the diagnosis of ALS, and therefore its treatment, and to improve quality of life, since sensory symptoms can be treated.

### Epidemiology and clinical features of patients with ALS

There is great heterogeneity in the prevalence of neuropathies in ALS studies due to heterogeneity in inclusion criteria and different diagnostic techniques used (in some studies EMG, in others sural biopsy or skin biopsy). The higher prevalence of neuropathies and sensory symptoms among ALS included in studies in which were performed sural and/or skin biopsies rather than EMG studies is probably due to a selection bias: biopsies are invasive exams and were performed more frequently in patients with clinical signs and/or symptoms of sensory neuropathy. Moreover, the lower prevalence of sensory symptoms or signs in the skin biopsy group compared to the sural biopsy group is harder to explain. It could be maybe due to the less frequent involvement of small fibers rather than large fibres, as reported by Hammad [7].

Sensory clinical signs and symptoms in ALS show a distal–proximal gradient; this is in line with the evidence of a length-dependent loss of sensory fibres observed in sural biopsy [7, 29, 38, 40] and in neurophysiological studies [6, 7, 9, 22].

Moreover, sensory impairment is usually concomitant with motor symptoms onset, and less frequently it precedes or follows them. This is important during the diagnostic process since the presence of sensory system alterations should not be an exclusion criterion for the diagnosis of ALS, as it may delay diagnosis and therapy.

No patients with bulbar onset of ALS included in case reports presented sensory symptoms. Most of the observational studies did not report clearly if ALS patients with sensory clinical signs and/or symptoms had a spinal or a bulbar onset. The only article in which a comparison between spinal vs bulbar onset ALS is reported, and in which authors did not show any difference in the incidence of sensory alterations between the two groups [24], included only patients with neurophysiological sensory alterations but without sensory clinical signs and/or symptoms.

Cognitive impairment was not associated with sensory alterations. Therefore, it seems that both cognitive alterations and sensory neuropathies are independent factors belonging to the non-motor spectrum of ALS.

Looking at therapies, only IVIG were revealed to be transiently effective. This might be explained by the coexistence of CIDP-like sensory neuropathy, whereas such a response would not be expected for ALS patients without signs of inflammatory neuropathy. More data need to be gathered on the efficacy of specific immunosuppressive and immunomodulating therapies in the treatment of sensory neuropathies associated with ALS and also on the efficacy of symptomatic therapies.

### Neurophysiological findings in ALS

NCS are a sensitive tool in the evaluation of sensory neuropathies in ALS patients. This is a relevant point because NCS are ordinarily performed in patients with the suspecion of ALS. It is important to be aware of these neurophysiological sensory alterations both for a correct early diagnosis of ALS and for better management of patients’ symptoms.

The presence of frequent neurophysiological features CIDP-like and the absence of other autoimmune comorbidities support the role of inflammation to justify these aspects, considering the growing evidence on immunity in ALS [45]. In these cases, the involvement of fast fibers cannot be excluded, but the exact cause remains unclear.

Finally, SSEPs can be altered in ALS patients, suggesting a possible impairment of the central sensory system, although only a few studies focused on this aspect.

### Neuropathological findings

The presence of a primary involvement of the sensory nervous system in ALS patients is supported by histopathological data. In particular, a progressive loss of both large and small sensory fibers was observed, in studies respectively evaluating sural nerve and skin biopsies. In one study [40] QST was demonstrated to be a good marker of histopathological evidence of sensory neuropathies. Therefore, due to the invasiveness of histopathological examinations, QST could be a useful surrogate of small fibres involvement in the evaluation of ALS patients, although more extensive studies are needed to be performed.

Although data of simultaneous explorations of small and large fibers are unfortunately unavailable, literature seems to support a double pathogenetic mechanism that is quite different from small and large fibers. While in small fibers a length-dependent degeneration similar to that recognized in second motor neurons can be hypothesized [46], in large sensory fibers an acute or subacute inflammatory process could be involved.

Even if these are just speculative observations, further studies and information about histopathologic findings could allow specific treatment strategies.

### Genetics

We did not identify, among the cases included in this review, any association between the incidence of sensory neuropathies in ALS patients and the presence of genetic mutations or polymorphisms in ALS causative genes. The only exception is represented by SOD1 mutated ALS patients, in which sensory neuropathy seems to be more frequent than other ALS subtypes. This association is supported by pre-clinical studies; in particular, it is known that mice models of SOD1 ALS have demonstrated the presence of neuropathy [47–49].

Moreover, juvenile ALS due to genetic mutations in SETX and SPTLC1 seems to be associated with an increased propensity to develop sensory neuropathies and dysautonomia.

Notably, some ALS patients also presented mutations in genes that are causative of neuropathy. In these cases, sensory neuropathy might be considered coexisting comorbidity due to other concomitant neurological diseases and not directly connected with ALS. In all these cases a stochastic association seems to be the most credible explanation.

### Limitations

Our study is not without limitations. A first possible methodological limitation is the inclusion of case reports, which is not common in systematic reviews. Given the expected paucity of data on the topic, we decided to maintain case reports and case series to gather further insight into aspects that have might be neglected in studies on larger samples, such as genetic features, progression of the disease, diagnostic delay, and possible treatments. However, we acknowledge that case reports present an inherent selection bias, and therefore the conclusions drawn from such studies need to be confirmed in larger studies. A second methodological limitation is that our estimate of the ALS diagnostic delay in the context of pre-existing neuropathy relies on only a fraction of the cases, as many papers do not report data useful to estimate diagnostic delay. Therefore, caution should be applied in the interpretation of the results.

There was great heterogeneity both in sensory neuropathy and ALS criteria used across different studies. For ALS diagnosis, most of the included studies used El Escorial criteria, which are complex, allow different levels of diagnostic certainty, and suffer from poor interrater reliability [50]. All of this could in theory limit the generalizability of our conclusion, due to the inclusion of ALS mimics (especially in the large observational studies, which often lack a pathological confirmation), and the exclusion of atypical ALS cases. The recently developed Gold Coast criteria might impact future studies on ALS and sensitive neuropathy, being more easy to apply and more sensitive to atypical ALS presentation [51].

Finally, it is possible that we did not see an association with cognitive impairment due to the fact that many studies were carried out at a time when this was considered an exclusion criterium for ALS. In other cases, cognition was simply not assessed.

## Conclusions

The findings of the observational studies and the case reports support the idea that ALS may also be characterized by subclinical or mild distal axonal neuropathy involving large-caliber myelinated fibers and small unmyelinated fibers as a part of the primary neurodegenerative process. The association between CIDP-like neuropathy and ALS needs to be better explored, although it could be interpreted as part of the neuroinflammatory process that seems to underlie ALS pathogenic mechanisms.

The presence of sensory neuropathy in ALS seems to be mostly associated with a spinal onset (in alignment with the proposed shared neurodegenerative mechanism of primary length-dependent axonopathy) and possibly with particular genetic forms of ALS (especially SOD1 and juvenile ALS, along with dysautonomia). Moreover, some studies suggest that it might be almost mutually exclusive with cognitive impairment.

All these data reveal the importance of searching for sensory neuropathy in ALS patients and can explain sensory symptoms frequently reported both in the early and advanced phases of the disease. Therefore, the early and correct identification of neuropathies-related symptoms is useful for improving their treatment and, consequently, patients’ quality of life. Regarding ALS diagnosis, we must continue to pay attention to differentiate ALS with sensory symptoms from other ALS-mimic conditions, but we should also remember that in patients with spinal ALS, a certain degree of sensory neuropathy, especially in neurophysiological studies and biopsies, can be present.

To solve this complex relationship, future multicenter longitudinal studies including ALS patients in the early phases of the disease and characterized by whole genotyping, deep clinical and neurophysiological phenotyping, and skin or nerve biopsy, are needed.

In conclusion, we suggest specifically including sensory neuropathy symptoms among non-motor symptoms of ALS.

## Data Availability

The data acquired in the preparation of the current work are available upon reasonable request to the corresponding author.
